# Improvement of Stress Resistance of Microencapsulated *Lactobacillus plantarum* by Emulsion Electrospinning

**DOI:** 10.3390/foods13121897

**Published:** 2024-06-17

**Authors:** Yuehan Wu, Shanshan Zhang, Ziyou Yan, Shiyang Li, Qianwen Wang, Zhiming Gao

**Affiliations:** 1 Cooperative Innovation Center of Industrial Fermentation (Ministry of Education & Hubei Province), Hubei University of Technology, Nanli Road, Wuhan 430068, China; yhwu@hbut.edu.cn; 2 Glyn O. Phillips Hydrocolloid Research Centre, School of Life and Health Sciences, Hubei University of Technology, Nanli Road, Wuhan 430068, China

**Keywords:** probiotics, core–shell, microencapsulation, viability, W/W emulsion

## Abstract

Probiotics have become increasingly recognized for their potential health-promoting properties; however, the viability of probiotics can be affected by storage and transportation processes as well as the stressful environment of the human digestive tract, preventing them from achieving effective concentration (10^7^ CFU/mL). In this regard, the embedding technology of probiotics provides an effective protection method. Dextran-based water in water (W/W) emulsion loaded with *Lactobacillus plantarum* was used as spinning solution to prepare *Lactobacillus plantarum*-loaded electrospun fibers. The structure of the W/W emulsion and the electrospun fibers was charactered. *Lactobacillus plantarum* were uniformly embedded in the internal phase of the W/W emulsion and the loading efficiency was 9.70 ± 0.40 log CFU/g. After 240 min digestion in the gastrointestinal tract, and temperature treatment in 65 °C and 72 °C, the loaded probiotics maintained high activity. Even after 5 days of storage in room temperature and 4 °C, the loaded probiotic activity levels remained high, with counts >8 log CFU/g. These results suggest that probiotics encapsulated by emulsion electrospinning could be potentially delivered in a novel food delivery system used in the future food industry.

## 1. Introduction

Probiotics, as beneficial microorganisms, have become increasingly recognized for their potential health-promoting properties. These live bacteria offer a myriad of benefits to human health, including improved digestion, enhanced immune function, and even mental well-being [1,2]. It has been reported that the viability of probiotics in a food matrix should be more than 10^7^ CFU/g to achieve sufficient health benefits [3,4,5,6,7]. However, the viability of probiotics is compromised by various factors during handling, storage, and passage through the gastrointestinal tract [8]. The acidic environment of the stomach, along with bile salts and enzymatic activity in the intestines, inhibiting the viability of probiotics, limits their efficacy upon reaching the gut [8,9]. To address this issue, researchers have explored various encapsulation techniques to safeguard probiotics during handling, storage, and passage through the gastrointestinal tract, with electrospinning emerging as a promising strategy [10,11,12].

Electrostatic spinning, a simple and convenient technology for producing micron-to-nanoscale fibers, offers a unique solution for encapsulating probiotics due to its large specific surface area and high porosity [13,14]. In conventional electrospinning, a polymer solution or melt is subjected to an electric field, resulting in the formation of continuous nanofibers deposited onto a collector substrate. In recent years, emulsion electrospinning has been rapidly developing to be an alternative technique of conventional electrospinning. With the rapid growth of new materials from renewable resources, diverse wall materials are appropriate for emulsion electrospinning, including biopolymers [15,16,17] (e.g., proteins, polysaccharides) and biocompatible polymers (e.g., poly vinyl alcohol (PVA [18]), polyethylene oxide (PEO) [19], polycaprolactone (PCL) [20]). They can be used individually or in combination according to the type and specific usages of the food ingredients.

One of the key advantages of emulsion electrospinning is its ability to produce core–shell nanofibers [20,21], the unique structure enables probiotics encapsulated within the core and surrounded by a protective shell of polymer material. The mechanism underlying the formation of core–shell nanofibers in emulsion electrospinning lies in the phase separation of the emulsion during the electrospinning process [22]. Emulsions typically consist of two immiscible liquid phases stabilized by surfactants or emulsifiers. When subjected to the electric field during electrospinning, the emulsion droplets elongate and stretch, leading to the evaporation of the solvent and the solidification of the polymer material. As the polymer solidifies, the emulsion undergoes phase separation, with the probiotics preferentially partitioning into the core phase, while the polymer forms the shell surrounding the core [23]. This core–shell structure provides enhanced protection for the encapsulated probiotics, shielding them from external factors such as pH changes and the enzymatic degradation encountered in the gastrointestinal tract [24].

Water-in-water (W/W) emulsion electrospinning represents a promising advancement in the field of probiotic encapsulation, offering several advantages over traditional oil-in-water emulsion electrospinning. Unlike oil-based emulsions, in which the oil phase may introduce potential allergens or interfere with body health, water-in-water emulsions are oil-free, inherently biocompatible and low-fat or fat-free, making them attractive for the consumer. These emulsions consist of two aqueous phases with different compositions, typically a polymer-rich phase and a probiotic-containing phase, stabilized by compatible surfactants or polymers [25,26]. Biopolymer-based water-in-water emulsions have potential applications in encapsulation and controlled release in the pharmaceuticals, food, and cosmetic industries [27]. These emulsion droplets offer great possibilities for developing innovative encapsulation and delivery systems for labile molecules in food formulations and functional foods. By using food-grade components, these delivery systems can be prepared through simple processing operations while ensuring biocompatibility [28,29]. Thus, water-in-water emulsion electrospinning presents a promising approach for encapsulating probiotics within core–shell nanofibers, offering enhanced protection and stability during transit through the gastrointestinal tract.

In this study, we successfully prepared the dextran-based nanofibers by W/W emulsion electrospinning; the physicochemical properties of W/W emulsion and the electrospun fibers were characterized. Furthermore, the electrospun fibers were used as encapsulants for *Lactobacillus plantarum*, and their ability to survive under in vitro gastrointestinal conditions and during storage was evaluated.

## 2. Materials and Methods

### 2.1. Materials

Dextran (Mw, 500,000), pepsin (3000 U/mg), trypsin (2500 U/mg) and RBITC were purchased from Shanghai Macklin Biochemical Co., Ltd. (Shanghai, China). Polyethylene oxide (Mw, 200,000) and LIVE/DEAD^®^ BacLight^TM^ Bacterial Viability Kit were purchased from Sigma-Aldrich (St. Louis, MO, USA). Sodium dihydrogen phosphate (NaH_2_PO_4_), potassium bromide (KBr), bile salts and ethanol were purchased from Sinopharm Chemical Reagent Co., Ltd. (Shanghai, China). The strain of *Lactobacillus plantarum* subsp. plantarum CICC 6240 (LP) was obtained from China Industrial Microorganism Collection and Management Center (Beijing, China). MRS broth was provided by Qingdao Hi–Tech Park Haibo Biotechnology Co., Ltd (Qingdao, China). Deionized water was used in all experiments.

### 2.2. Preparation of W/W Emulsion

The stock solutions of polyethylene oxide (15 wt%) and dextran (15 wt%) were prepared by dissolving in deionized water under constant stirring using a mechanical stirrer until homogeneous at 20 °C. The W/W emulsion was prepared by mixing a certain amount of PEO and DEX stock solution using a magnetic stirrer at 600 rpm for 3 min. The finally obtained emulsion composition was composed of different concentrations (1 wt%, 2 wt%, 3 wt%, 4 wt%, 5 wt%, 6 wt%, 7 wt%, 8 wt%, 9 wt%, 10 wt%,) of PEO with 10% DEX, and named as 1%PEO/10%DEX, 2%PEO/10%DEX, 3%PEO/10t%DEX, 4%PEO/10%DEX, 5%PEO/10%DEX, 6%PEO/10%DEX, 7%PEO/10%DEX, 8%PEO/10%DEX, 9%PEO/10%DEX, 10%PEO/10%DEX, respectively (Table S1). For the encapsulation of probiotics, *Lactobacillus plantarum* was added in the DEX phase, and the final concentration of *Lactobacillus plantarum* in the emulsion was 10^9^ CFU/mL.

### 2.3. Electrospinning Process

The electrospinning device (Figure S1) was placed horizontally, using a 5 mL syringe connected with a 19G needle with an inner diameter of 0.67 mm, and the injection rate was 0.5 mL/h. The voltage of the high-voltage DC power supply was 15 kV, and the distance between the needle and the tin collector was 15 cm. Throughout the experiment, the temperature and relative humidity were controlled at 25 ± 2 °C and 50 ± 5%, respectively, and the air bubbles inside the needle were discharged before the experiment.

### 2.4. Characterization

Confocal images were performed using a confocal laser-scanning system (Leica microsystems Inc., Heidelberg, Germany), dextran was attained with RBITC and the probiotics were stained by LIVE/DEAD^®^ BacLightTM Bacterial Viability Kit. Rheological measurements were performed using a HAAKE Rheostress 6000 rheometer (Thermo Scientific, Franklin, Germany) with a 60 mm diameter double cone/plate sensor (C60/1°Ti L). The flow behavior (shear stress, shear rate) was recorded at 25 ± 0.5 °C and 60 °C, respectively, in controlled shear mode using steady-state conditions between 0.01 and 1000 s^−1^. The conductivity of zein solutions was measured using a benchtop conductivity tester at room temperature and expressed in μS/cm.

The morphology of electrospun fibers were collected using a scanning electron microscopy (SEM; JSM-6390LV, Tokyo, Japan) after sputtering with a gold-palladium mixture in a vacuum. We randomly selected 100 individual fibers and analyzed their diameters using ImageJ software (verion 1.54c). The core–shell structure was characterized by transmission electron microscopy (TEM, JEM-2100 F, Tokyo, Japan). The conductivity of zein solutions were using a benchtop conductivity tester at room temperature and expressed in μS/cm.

Fourier-transform infrared spectroscopies (FT-IR) of the samples were recorded at an ambient temperature on an FT-IR spectroscopy (Thermo Fisher Scientific, Nicolet iS10, Waltham, MA, USA) in transmittance mode in the range of 4000–400 cm^−1^; the wavenumber resolution and scan number for all samples were 4 cm^−1^ 64 scans, respectively. The electrospun films were cut into small pieces and dried overnight in an oven at 40 °C, then mixed and ground into powders with potassium bromide (KBr); the mixture was pressed to form a sample disk for the tests.

To prepare simulated gastric juice, 100 mL of ultrapure water was taken, and the pH was adjusted to 2.5. The solution was then sterilized using a high-pressure sterilizer before adding 330 mg of pepsin enzyme. Subsequently, the mixture was filtered through a Millipore filter with a pore size of 0.22 μm. To prepare simulated intestinal fluid, another batch consisting of an additional 100 mL portion of ultrapure water was obtained and combined with precisely 1.36 g potassium dihydrogen phosphate and 300 mg bile salts. The pH was adjusted to 6.8 before sterilization. Following that, exactly 1 g of pancreatic enzyme was introduced into the solution before filtration using a Millipore filter with a pore size of 0.22 μm.

The probiotic loaded electrospun fiber mat was cut into a disk with a diameter of 10 mm, and then shaken in a PBS buffer for 30 min to obtain probiotic suspensions. The survival probiotic was enumerated by the drop plate method. The bottom of the agar plates was divided into four quadrants and labeled, and 100 μL of each serial dilution was placed into one quadrant of each plate. The survival probiotics on the agar plate were enumerated after incubation for 24 h.

### 2.5. Statistical Analysis

The results were performed for at least three samples (*n* ≥ 3) to confirm the reproducibility of the experimental result, and were expressed as mean ± standard deviation. The results were evaluated using one-way ANOVA. A value of *p* < 0.05 was considered statistically significant.

## 3. Results and Discussion

### 3.1. Characterization of W/W Emulsions

**Figure 1:**
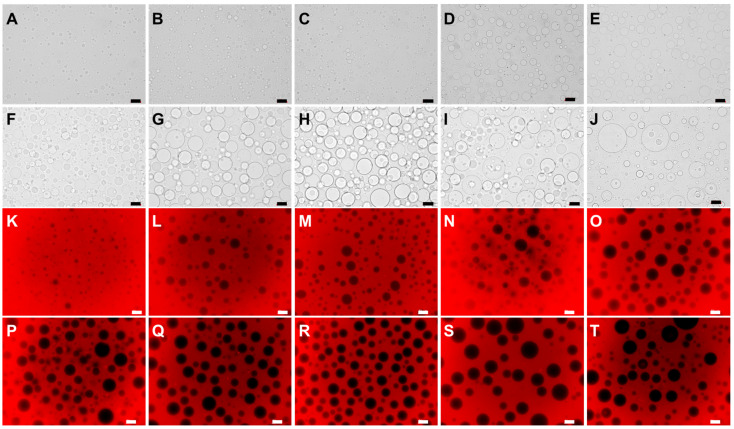
(**A**–**J**) Microscope image of PEO/DEX W/W emulsion, 10 wt% PEO and 1–10 wt% DEX. (**K**–**T**) Fluorescence microscope image of PEO/DEX W/W emulsion, 10 wt% PEO and 1–10 wt% DEX; red area is rich in RBITC-labeled DEX, black area is rich in PEO. The scale is 50 μm.

The optical structure of the W/W Pickering emulsions prepared with different dextran concentrations is shown in Figure 1A–J; the droplet size of the W/W emulsions tends to increase with an increase in the DEX concentration. The droplet size increases from 14.92 ± 4.49 μm to 73.77 ± 23.73 μm when the DEX concentration increases from 1 wt% to 10 wt%. This may be due to the increased external phase concentration, exacerbating the repulsion between the two phases, thereby increasing the aggregation of the internal phase, and consequently, increasing the droplet size [30].

In order to investigate the distribution of phases in the PEO/DEX W/W emulsion system, dextran was stained by RBITC. As shown in Figure 1K–T, the red area represents the DEX phase marked with RBITC and the black area represents the PEO phase. It can be seen that DEX remains as the continuous phase despite the increase in the concentration from 1 wt% to 10 wt%. No phase inversion phenomenon is observed with the increase in the PEO concentration, which might be due to the larger molecular weight of the DEX forming such a W/W emulsion system.

**Figure 2:**
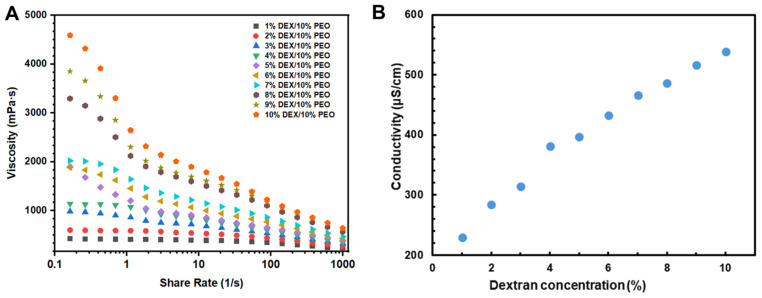
The rheological properties (**A**) and conductivity (**B**) of W/W emulsions with different dextran concentrations.

Viscosity is one of the key factors in the phase separation of emulsions; meanwhile, during the electrospinning process, the viscosity of the spinning solution is also one of the key factors affecting the fibers’ structure [31,32]. The lower viscosity may prevent electrospinning and result in the formation of electrostatic spray particles rather than electrospun fibers [33]. In our previous study, even slight changes in viscosity could have significant effects on the properties of nanofibers, causing either subtle or significant changes [13]. For this reason, viscosity tests were conducted on three W/W emulsion systems, and the viscosity curve as a function of shear stress was obtained. As shown in Figure 2A, the viscosity of the emulsions decreases with increasing shear stress, demonstrating a shear-thinning behavior. It is noteworthy that the viscosity increases with the total concentration, as increased concentration leads to more entanglement of molecules, increasing the viscosity of the samples. The concentration of the solution is a key factor affecting the entanglement state of polymer chains, which is crucial for maintaining the stability of polymer chains in the jet during electrospinning [14].

The electrical conductivity of spinning solutions is considered a crucial parameter in the electrospinning process. The formation of jets is directly related to the movement of charged polymer solutions toward a grounded target; researchers found that low conductivity reduced the spinnability of solutions [34]. The conductivity of PEO/DEX W/W emulsions was measured by a desktop conductivity meter. As shown in Figure 2B, the conductivity of PEO/DEX W/W emulsions increases with the increased concentration of DEX, which might be due to the higher concentrations of DEX containing more ions or charged substances that can increase conductivity in water. A high concentration of dextran solutions means closer molecular distances and enhanced interactions among solutes, leading to easier electron or ion conduction, thereby increasing conductivity.

### 3.2. Characterization of Electrospun Fibers

**Figure 3:**
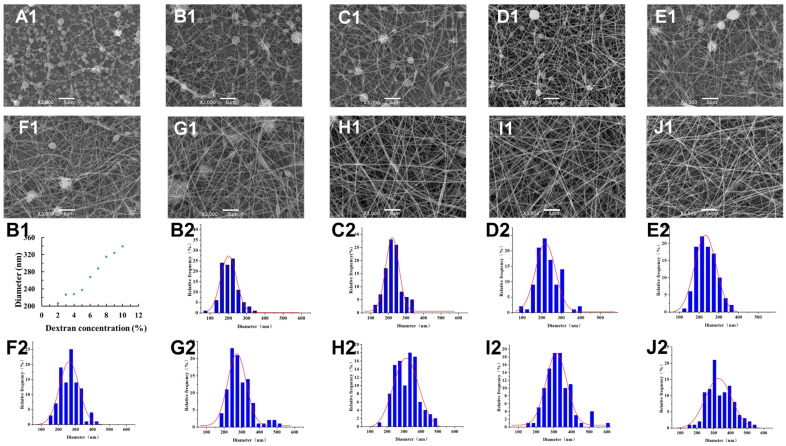
(**A1**–**J1**) SEM image of electrospun fiber prepared by W/W emulsion consisting of 10 wt% PEO and 1–10 wt% DEX, respectively, with a scale of 5 μm; (**B1**) the diameter of electrospun fiber prepared by W/W emulsion with different dextran concentrations; (**B2**–**J2**) the diameter distribution of electrospun fiber prepared by W/W emulsion consisting of 10 wt% PEO and 2–10 wt% DEX, respectively.

Different concentration ratios of W/W emulsions with varying properties of solution might affect electrospun fiber properties. Scanning electron microscope was used to observe the morphology of fibers electrospun by the W/W emulsion. As shown in Figure 3A1–J1, when electrospun, the emulsion consists of 10 wt% PEO and 1 wt% DEX, and the fibers are mostly bead-on-string fibers. As the DEX concentration increases further, the fiber morphology transitions from irregular bead-on-string to spindle fibers, and finally to uniform beadless fibers. The increasing concentration of the dextran concentration increases the entanglement between molecules, thus increasing the viscosity. This higher viscosity allows the fibers to resist the forces in the electric field more effectively, making them less likely to break. At lower concentrations, the entanglement is insufficient to withstand the pulling forces in the electric field, leading to the formation of irregular fibers [13].

The diameter of nanofibers affects their application in the food industry [35]. Larger fiber diameters can provide more internal space, and usually have higher mechanical strength and tensile properties, suitable for applications requiring higher strength [36]. Furthermore, larger diameter electrospun fibers can hold more payload substances, facilitating controlled release rates. Smaller diameter fibers have a larger surface area ratio, which enhances their contact with the environment, improving reaction efficiency and adsorption performance. Therefore, controlling fiber diameter size is an explored means of fabrication. The diameter of the electrospun fibers was measured using Image J software, and the result was shown in Figure 3A1–J1. The bead-on-string fibers electrospun by the emulsion consisting of 10 wt% PEO and 1 wt% DEX were uneven and fragile, which made it hard to collect their diameter. For the fibers electrospun by the emulsion consisting of 10 wt% PEO and 2–10 wt% DEX, the average diameter of nanofibers increased with an increase in the DEX concentration. For example, when the DEX concentration was 2 wt%, the average fiber diameter was 207.09 nm, and when the concentration increased to 10 wt%, the diameter increased to 338.90 nm. This increase occurred because, as mentioned earlier, increased concentration and viscosity mean that, under the same voltage, the fibers experience the same pulling force, resulting in an increase in fiber diameter [36,37].

**Figure 4:**
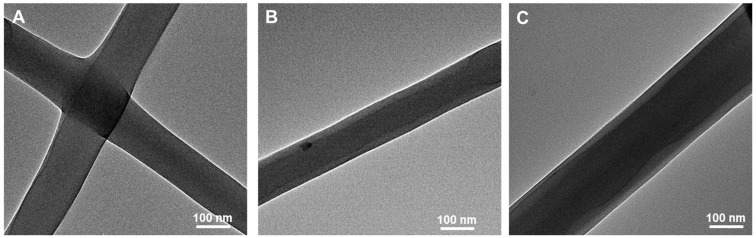
The TEM image of fiber electrospun by 5%DEX/10%PEO (**A**), 7%DEX/10%PEO (**B**) and 10%DEX/10%PEO (**C**).

In order to verify whether the prepared nanofibers are of a core–shell structure, transmission electron microscopy was used to verify the structure of the electrospun fibers. The color differences in the TEM images mainly reflect the differences in density within the sample. In electrospun fibers, the nanofiber shell and core have different densities. Since the double-layer fibers at the center of the fiber have a denser structure, they appear darker; the outer layer, having only a single fiber layer, is less dense, and thus, appears lighter. This color difference allows for a visual confirmation of the formation of core–shell structured nanofibers. In Figure 4A, when the PEO concentration is 10 wt% and the DEX concentration is 5 wt%, the core–shell structure is not fully formed, which, possibly due to the lower viscosity at low concentrations, makes the inner phase more likely to break under pulling forces. In Figure 4B,C, the emulsion of electrospun fibers is of a core–shell structure; as the DEX concentration increases, the overall concentration and viscosity of the system increase, leading to uniform and distinct core–shell structures. The TEM images of electrospun fibers show clear demarcation lines, indicating a well-defined core–shell structure. Also, as the DEX concentration increases, the diameter of the core fibers also increases proportionally.

**Figure 5:**
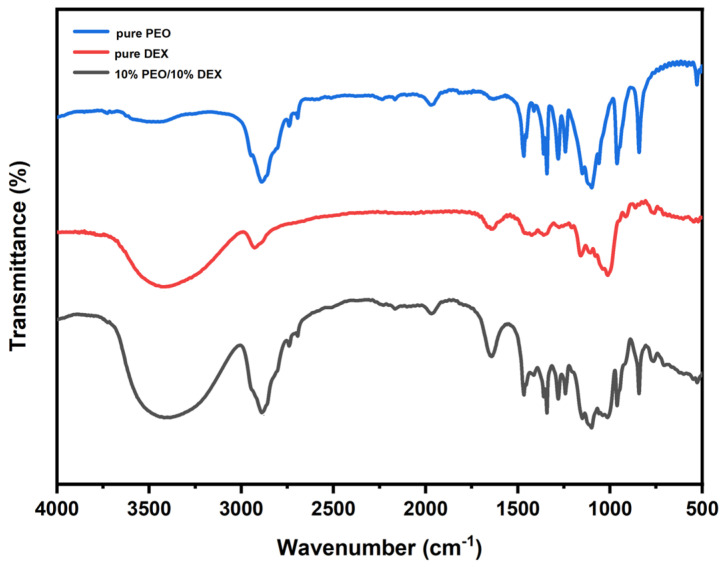
The FT-IR spectra of pure PEO powder, pure DEX powder and emulsion electrospun fibers [17].

The FT-IR spectra of nanofibers are shown in Figure 5; according to the spectra, forming W/W emulsions followed by electrospinning does not cause chemical reactions between the two phases. The FT-IR spectra of W/W emulsions at different concentration ratios show no significant changes. For pure DEX, the presence of –OH groups results in bands at 3000–3700 cm^−1^; bands due to C–H stretching appear at 2906 cm^−1^, and bands due to C–H bending and wagging vibrations occur at 1417 cm^−1^ and 917 cm^−1^. A broad band observed from 1185 to 1135 cm^−1^ with a main peak at 1162 cm^−1^ is due to asymmetric ring C–O–C stretching [38]. In the FT-IR spectra of pure PEO, the characteristic peak of PEO appears near 2889 cm^−1^, attributed to CH_2_ stretching, with other characteristic bands observed at 961 cm^−1^ and 842 cm^−1^ related to C–O–C stretching vibrations. Sharp peaks attributed to methyl CH deformation are observed at 1341, 1282, 1242, and 1154 cm^−1^ [39]. In PEO/DEX W/W emulsion systems, aside from some characteristic peaks of PEO and DEX, no new sharp peaks appear, but the positions of peaks shift, moving to lower wavenumbers, suggesting that the mixing of the two substances causes some interactions [40]. This confirms the uniformity and homogeneity of the prepared nanofibers and the stability of the electrospinning process.

### 3.3. Structure of Lactobacillus plantarum-Loaded Electrospun Fibers

**Figure 6:**
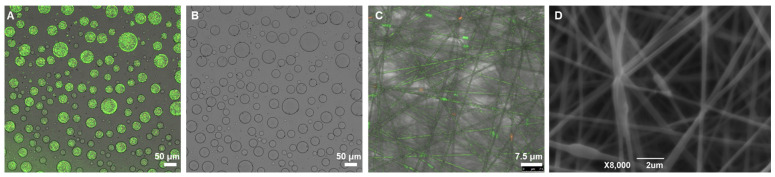
CLSM image of 7%DEX/10%PEO emulsion loaded with *Lactobacillus plantarum*, (**A**) dark fields, and (**B**) bright field. CLSM image (**C**) and SEM image (**D**) of 7%DEX/10%PEO electrospun fiber loaded with *Lactobacillus plantarum*.

To explore the preference of *Lactobacillus plantarum* for the internal and external phases of the PEO/DEX W/W emulsion systems, the bacteria were labeled by LIVE/DEAD^®^ BacLight^TM^ Bacterial Viability Kit and then incorporated into W/W emulsions. The emulsions were then observed under a confocal laser scanning microscope. As shown in Figure 6A,B, when the PEO concentration is 10 wt% and the DEX concentration is 7 wt%, after adding a certain amount of labeled *Lactobacillus plantarum* to the emulsion, *Lactobacillus plantarum* was uniformly embedded in the internal phase of the W/W emulsion and was present in large numbers. At this concentration ratio, based on a previous result in Figure 1Q, DEX acts as the external phase and PEO as the internal phase, so the higher molecular weight and viscosity of DEX compared to PEO allow for the uniform distribution of probiotics in the internal phase.

To preliminarily explore the embedding effect of the emulsion electrospun fiber, the labeled *Lactobacillus plantarum* was mixed with the emulsion and electrospun under appropriate conditions. As shown in Figure 6C, the electrospinning of a 10 wt% PEO and 7 wt% DEX concentration ratio effectively encapsulates the probiotics within the nanofibers; the overlay image shows that the bacteria are completely wrapped inside the nanofibers. Combining the fluorescence microscope and CLSM images of the W/W emulsion-embedded probiotics confirms that regardless of the phase preference of the bacteria within the emulsion system, after electrospinning, the probiotics are encapsulated within the electrospun fibers.

In order to further verify whether the W/W emulsion electrospinning systems had indeed encapsulated the probiotics, an SEM image was collected. In Figure 6D, it can be seen that, although the diameter of the probiotics is larger than that of the nanofibers, they are still completely encapsulated within the fibers. *Lactobacillus* cells orient along the nanofibers during the Taylor cone process in electrospinning, with the polymer solution containing randomly oriented bacteria initially aligning mainly along the streamlines and further arranging themselves within the jet, eventually solidifying into nanofibers. SEM observations, combined with confocal and fluorescence microscopy (Figure 6C), show that *Lactobacillus plantarum*, as either individual or dividing cells, is uniformly distributed within the nanofiber mats.

### 3.4. Survival ability of Lactobacillus plantarum-Loaded Electrospun Fibers

The encapsulated ratio of the *Lactobacillus plantarum*-loaded electrospun fibers was measured by the drop plate method. After the electrospinning process, the viable probiotics load in the electrospun fiber was 9.70 ± 0.40 log CFU/g, which was much higher than the effective concentration (7 log CFU/g). The release mechanisms of probiotics from the microstructure probably involve the gradual breakdown of the core–shell structure, ensuring a controlled release of probiotics into the gastrointestinal tract.

**Figure 7:**
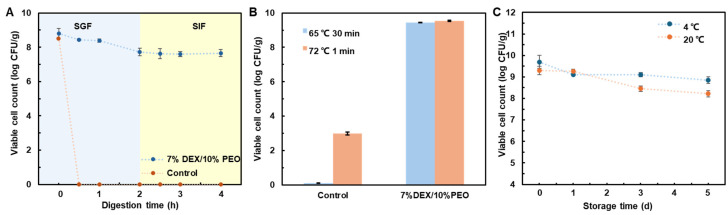
(**A**) Changes in the number of probiotics in 7%DEX/10%PEO electrospun fiber after simulated gastrointestinal digestion. (**B**) Changes in the number of probiotics in 7%DEX/10%PEO electrospun fiber after heat treatment. (**C**) Changes in the number of probiotics in 7%DEX/10%PEO electrospun fiber during a 5-day storage.

The viability of probiotics is compromised by various factors when passing through the gastrointestinal tract. The acidic environment of the stomach, along with bile salts and enzymatic activity in the intestines, inhibits the viability of probiotics. To explore the survival ability of probiotics encapsulated in W/W emulsion electrospinning systems during gastrointestinal digestion, an in vitro simulated gastrointestinal digestion was conducted. During the experiment, probiotic suspension drops which were marked as the control were added to simulated gastric fluid and immediately diluted and counted. As shown in Figure 7A, when untreated probiotics were exposed to the simulated gastric fluid, it was found that the acidic environment of the stomach severely damaged the probiotics. After 60 min of digestion in the simulated gastric fluid, dilution and counting detected no bacterial colonies, confirming that free *Lactobacillus plantarum* could not effectively resist the damage from strong stomach acids. This highlights the significant impact of the acidic environment in the stomach on probiotics and emphasizes the necessity of protective measures to ensure their survival and function in the gastrointestinal tract. After 120 min of digestion in gastric fluid, the count drops from 8.79 ± 0.30 log CFU/g to 7.73 ± 0.22 log CFU/g after 120 min, a decrease of one order of magnitude. Then, after 120 min of digestion in simulated intestinal fluid, the number of surviving probiotics is 7.66 ± 0.20 log CFU/g, nearly unchanged from the initial count. The probiotics loaded on the 7%DEX/10%PEO electrospun fiber survive; after 240 min of digestion, the number of active probiotics shows a significant difference from the initial 0 h but still maintains high activity. The results indicate that electrospinning probiotics into W/W emulsions can effectively protect them during exposure to gastrointestinal fluids, potentially offering controlled release effects that allow the probiotics to continuously benefit the gut without being affected by stomach acids. The encapsulation of probiotics within core–shell structured fibers helps protect them from the harsh acidic environment in the stomach, supporting their survival.

The viability of probiotics is compromised during industry handling; one of the key factors is temperature treatment. During the temperature treatment process, the probiotics’ activity significantly decreases at high temperatures. Therefore, to explore whether the emulsion electrospun fibers can protect probiotics under high temperature conditions, a heat stability experiment was conducted. Both untreated probiotics and electrospun probiotic-loaded fibers (9.70 ± 0.40 log CFU/g) were subjected to a 65 °C treatment for 30 min and a 72 °C treatment for 1 min. As shown in Figure 7B, untreated probiotics lose viability after 30 min at 65 °C; those encapsulated in nanofibers maintain a high level of activity (9.94 ± 0.11 log CFU/g), while the control group, which did not receive encapsulation, shows a count of 0. Similarly, after 1 min at 72 °C, the number of viable bacteria in untreated samples was only 2.99 ± 0.09 log CFU/g, whereas probiotic-loaded fibers still show high activity at 9.54 ± 0.04 log CFU/g. It is speculated that the electrospun fibers may form a protective barrier that effectively shields the probiotics from external environmental influences [41]. This barrier could help maintain their activity by slowing down the heat transfer to the probiotics, possibly also due to the stable microstructure of the electrospun fibers, which encapsulates and secures the probiotics internally, preventing direct damage from high temperatures [42]. This structure likely helps in maintaining probiotic activity, and the lower moisture content might also play an important role.

A minimum required threshold of live probiotic cells in food should be 10^6^–10^7^ CFU/mL or grams of food to reach the effect concentration. However, their viability, and thereby their bioactivity, could be significantly compromised during storage, maintaining viability over long storage periods is of great important in the food industry. Thus, experiments were conducted to test the storage stability of probiotic-loaded fibers electrospun from PEO/DEX W/W emulsions and stored under conditions of 20 °C and 4 °C. The result is shown in Figure 7C; probiotics loaded on emulsion electrospun fibers maintain probiotic activity over time, whether stored at room temperature or 4 °C. Even after 5 days of storage in room temperature, probiotic activity levels remain high, with counts >8 log CFU/g, indicating that the concentration of active probiotics is sufficient. During 4 °C storage, the number of viable probiotics decreases by less than an order of magnitude and remains at a high level; at room temperature, the change is only by one order of magnitude. This suggests that at 4 °C, the probiotics are in a dormant state, hence the observed difference.

## 4. Conclusions

A W/W emulsion was prepared by dextran as the inner phase and polyethylene oxide as the outer phase. After electrospinning the W/W emulsion, the diameter of the obtained fibers increased as the dextran concentration increased; when the dextran concentration increased to 5%, the fiber gradually appeared to have a core–shell structure. After being loaded with *Lactobacillus plantarum*, the probiotics remained in the inner phase, and the loading efficiency was 9.70 ± 0.40 log CFU/g. After 240 min of digestion in the gastrointestinal tract, and temperature treatment in 65 °C and 72 °C, the loaded probiotics maintained high activity. Even after 5 days of storage in room temperature and 4 °C, the loaded probiotic activity levels remained high. These results indicate that the electrospun fibers may form a protective barrier that effectively shields the probiotics from the gastrointestinal tract and external environmental influences. These results suggest that probiotics encapsulated by emulsion electrospinning could be potentially delivered in a novel food delivery system used in the future food industry.

## Data Availability

The original contributions presented in the study are included in the article/Supplementary Material, further inquiries can be directed to the corresponding author.

## Supplementary Materials

The following supporting information can be downloaded at: https://www.mdpi.com/article/10.3390/foods13121897/s1, Figure S1: The illustrating of electrospinning process.; Table S1: The preparation formula of W/W emulsion.
