# Supplementary material for: Improvement of Stress Resistance of Microencapsulated Lactobacillus plantarum by Emulsion Electrospinning
Source: Foods. 2024 Jun 17;13(12):1897. doi: 10.3390/foods13121897 (PMC11203365; doi:10.3390/foods13121897)
Supplement: Supplementary file 1 [file foods-13-01897-s001.zip › foods-3031902-supplementary.pdf]

**Table S1.** The preparation formula of W/W emulsion.

| Sample | PEO Concentration (wt%) | Dextran Concentration (wt%) |
|---|---|---|
| 1%PEO/ 10%DEX | 1 | 10 |
| 2%PEO/ 10%DEX | 2 | 10 |
| 3%PEO/ 10%DEX | 3 | 10 |
| 4%PEO/ 10%DEX | 4 | 10 |
| 5%PEO/ 10%DEX | 5 | 10 |
| 6%PEO/ 10%DEX | 6 | 10 |
| 7%PEO/ 10%DEX | 7 | 10 |
| 8%PEO/ 10%DEX | 8 | 10 |
| 9%PEO/ 10%DEX | 9 | 10 |
| 10%PEO/ 10%DEX | 10 | 10 |

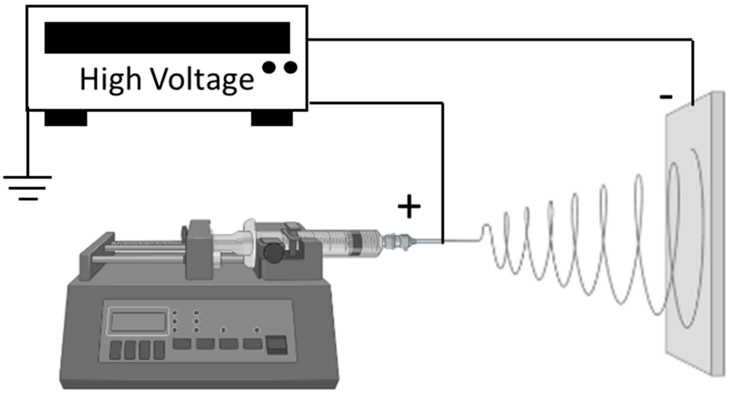


**Figure S1**. The illustrating of electrospinning process.
